# Leadership and Job Demands-Resources Theory: A Systematic Review

**DOI:** 10.3389/fpsyg.2021.722080

**Published:** 2021-09-30

**Authors:** Lars G. Tummers, Arnold B. Bakker

**Affiliations:** ^1^School of Governance, Utrecht University, Utrecht, Netherlands; ^2^Center of Excellence for Positive Organizational Psychology, Erasmus University Rotterdam, Rotterdam, Netherlands

**Keywords:** leadership, job resources, job demands, systematic review, job demands-resources theory

## Abstract

The purpose of this article is to provide a systematic review of leadership and Job Demands-Resources (JD-R) theory. We have analyzed 139 studies that study the relationship between leadership and Job Demands-Resources (JD-R) theory. Based on our analysis, we highlight ways forward. First, research designs can be improved by eliminating endogeneity problems. Regarding leadership concepts, proper measurements should be used. Furthermore, we point toward new theory building by highlighting three main ways in which leadership may affect employees, namely by: (1) directly influencing job demands and resources, (2) influencing the impact of job demands and resources on well-being; and (3) influencing job crafting and self-undermining. We hope this review helps researchers and practitioners analyze how leadership and JD-R theory can be connected, ultimately leading to improved employee well-being and organizational performance.

## Introduction

“*Inspirational leadership as a work resource might lead to positive attitudes, such as happiness at work.”* Salas-Vallina and Fernandez (2017, p. 628)

“*[I]n line with a job demands-resources perspective […], destructive forms of leadership […] may be perceived as demands or stressors that increase the propensity to drink among subordinates.”* Nielsen et al. (2018, p. 575)

“*Transformational leadership behaviors could play a more distal role than work organization factors by acting simultaneously on perceived job resources and job demands.”* Fernet et al. (2015, p. 27)

The three quotes show that leadership is essential for employee well-being and performance (see also Antonakis and Day, 2017). They also show that scholars link leadership to Job Demands-Resources (JD-R) theory. The Job Demands-Resources (JD-R) theory is often used to analyze how the work environment affects well-being and performance (Bakker and Demerouti, 2017). The third point—and the one we want to emphasize—is that the quotes show that scholars connect leadership and JD-R theory in various ways. Some see leadership as a job resource (quote 1), others as a job demand (quote 2), and still others see leadership not as a job resource or a job demand but as a factor influencing job demands and resources (quote 3).

However, quotes are not always representative of the literature. We need a thorough understanding of how leadership and JD-R theory can be connected. This can be done via a structured overview of the literature. To date, such an overview is lacking. We, therefore, present a systematic literature review. A systematic review analyzes the current body of knowledge in a transparent and reproducible way. We adhere to the widely used “Preferred Reporting Items for Systematic Reviews and Meta-Analyses” (PRISMA), ensuring transparent and complete reporting (Liberati et al., 2009). The PRISMA checklist is shown in Appendix 1.

We aim to answer three research questions (RQs). RQ1 focuses on the research methods and designs used by scholars who study leadership and JD-R theory. We then discuss which leadership concepts scholars have used, including transformational leadership, servant leadership, and Leader-Member Exchange (LMX) (RQ2). We end by analyzing how scholars have connected leadership to the various elements of JD-R theory (job demands, job resources, personal resources, and job crafting). Hence, we aim to answer the following three research questions:

- Which research methods and designs have been used to analyze the leadership-JD-R relationship?
- Which leadership concepts have been used—and which have been ignored—when studying the leadership-JD-R relationship?
- How has the relationship between leadership and JD-R theory been conceptualized?

Our goal for this review is to offer an agenda for future research on leadership and JD-R theory. This agenda aligns with the research questions. First, by studying the research methods and designs that scholars have used to analyze the leadership-JD-R relationship, we will show how scholars can improve methods and designs in future studies. Among others, we highlight that research designs can be improved by eliminating endogeneity problems (Antonakis et al., 2010).

Second, regarding leadership concepts, our review shows that scholars often use leadership concepts like transformational leadership and LMX. There are substantial critiques regarding these concepts, especially how they are currently measured (Van Knippenberg and Sitkin, 2013; Gottfredson et al., 2020). Sometimes these concepts can be helpful, but proper measurements should be used. For instance, high-quality measurement instruments of transformational leadership have been developed (Jensen et al., 2019). We urge scholars to analyze these critiques and the solutions in detail.

Third, by analyzing how scholars have conceptualized the relationship between leadership and JD-R theory, we show that the current way makes it hard to develop cumulative knowledge. Scholars have connected leadership and JD-R theory in many ways. This makes it hard to build upon each other's work. We also move beyond simply summarizing what scholars have done. In addition, we provide a new theoretical perspective on how leadership and JD-R theory can be connected. We contribute to theory building by highlighting three main ways in which leadership may affect employees, namely by (a) directly influencing job demands and resources, (b) influencing the impact of job demands and resources on well-being (strain and motivation); and (c) influencing job crafting. Future studies could use these three main ways to study the connections between leadership and JD-R in depth. In this way, we hope our review helps structure the knowledge on leadership and JD-R theory and provide theoretical ways forward (Short, 2009).

## A Background on JD-R Theory and Leadership

### Job Demands-Resources Theory

**Figure 1:**
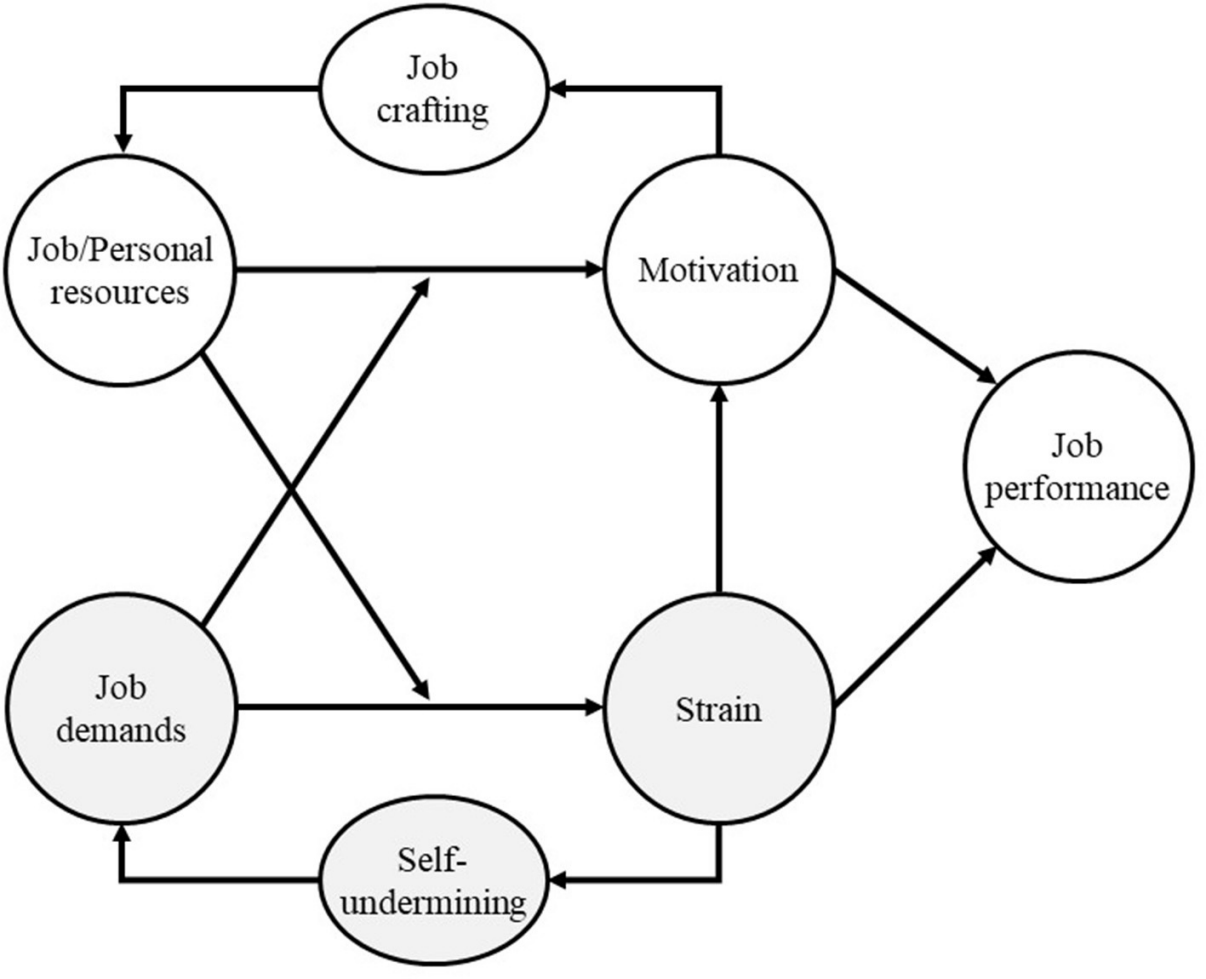
Job Demands–Resources (JD-R) theory, adapted from Bakker and Demerouti (2017).

Job Demands–Resources (JD-R) theory (Figure 1) explains how the organizational environment impacts employee well-being and performance. We shortly discuss the core aspects of the theory (for detailed discussions, see Demerouti et al., 2001; Bakker and Demerouti, 2007, 2017). A central proposition in JD-R theory is that although employees work in various sectors—such as academia, manufacturing, transport, or finance—their job characteristics can be classified into two categories: job demands and job resources.

Job demands are job aspects that require sustained effort and are, therefore, associated with physiological and psychological costs (Demerouti et al., 2001; Bakker and Demerouti, 2017). Examples include having a high workload, experiencing conflicting demands from managers and clients, and bullying. Job resources refer to aspects of the job that help reach work-related goals, reduce job demands and the associated costs, and stimulate personal growth and development (Demerouti et al., 2001). Examples are social support from your colleagues, having the freedom to decide whether to work at home or the office, and having opportunities to be promoted.

The JD-R theory has developed since the publication of Demerouti et al. (2001). One extension was including personal resources in JD-R theory (Bakker and Demerouti, 2007; Xanthopoulou et al., 2009). Personal resources refer to people's beliefs about how much control they have over their environment (Bakker and Demerouti, 2017). Examples are self-efficacy and optimism.

A fundamental proposition of JD-R theory is that job demands and job and personal resources activate different processes (Demerouti et al., 2001). Job demands can lead to a health-impairment process: having high job demands—such as an extreme workload—leads to constant overtaxing and, in the end, to burnout. Burnout happens when “one is cynical about the value of one's occupation and doubtful of one's capacity to perform” (Maslach et al., 1996, p. 20). In contrast, resources lead to a motivational process: having high job resources leads to more motivation, resulting in increased work engagement. Work engagement is the mental state where people feel energetic (vigor), are enthusiastic about their work (dedication), and are so immersed in their work that time seems to fly (absorption) (Bakker and Demerouti, 2017). In the end, job strain—shown by being burned out—leads to lower job performance. In contrast, motivation—shown by being engaged—leads to higher job performance.

Job demands and resources also interact to predict strain and motivation. In their article in 2001, Demerouti et al. already hinted at the possibility of such interaction. However, given that there was at that time little empirical evidence, they concentrated on the main effects. Later studies have provided evidence of the interaction effects (Bakker et al., 2005; Dicke et al., 2018). For instance, if you have much autonomy in your work (a job resource), this helps you deal with a high workload (a job demand). In this way, job autonomy reduces the adverse effects of a high workload.

JD-R theory has also incorporated two self-reinforcing paths (Bakker and Demerouti, 2017). A positive self-reinforcing path—or gain spiral—involves job crafting. People craft their jobs when they proactively change their job demands and resources (Wrzesniewski and Dutton, 2001; Tims et al., 2012). For instance, a junior scholar can increase her job resources by asking for regular feedback from her supervisor. These increased job resources lead to even higher motivation, thereby restarting the positive self-reinforcing path, or “gain spiral” (Hobfoll, 1989; Van Wingerden et al., 2017).

However, not all self-reinforcing paths are positive. Scholars highlighted the negative self-reinforcing path—or loss spiral—known as self-undermining. Self-undermining is “behavior that creates obstacles that may undermine performance” (Bakker and Costa, 2014, p. 115). Say that an accountant experiences burnout. Because of this burnout, he starts making mistakes. As his work quality diminishes, his director asks him to think about a plan to improve his work. This additional task increases his workload, restarting the self-undermining cycle (Bakker and Wang, 2020).

### Leadership

Every organization needs leadership to solve coordination problems: someone has to decide—alone or with others—what the organization's strategy is, whom to hire, whom to fire, and how conflicts between staff members or between staff and clients can be resolved. We define leadership as an influencing process, specifically an intentional influence to guide, structure, and facilitate others. Current definitions align with this, highlighting that leadership is an intentional influencing process (Dansereau et al., 2013; Yukl, 2013; Antonakis and Day, 2017).

Leadership is vital for employee well-being and performance (Dinh et al., 2014; Antonakis and Day, 2017). Based on a meta-analysis of primarily surveys, Judge and Piccolo (2004) concluded that the correlations between transformational leadership and employee job satisfaction and employee motivation were positive. Experimental studies showed that leadership affects employee well-being and performance (Barling et al., 1996; Dvir et al., 2002; Bellé, 2014). For instance, in a recent study, Chemin (2021) showed that high-ability and hard-working leaders increase employee effort, knowledge-sharing, and performance.

As noted, leadership is also increasingly linked to JD-R theory (Syrek et al., 2013; Perko et al., 2014; Diebig et al., 2017; Cheung et al., 2021). Many scholars who study leadership and JD-R theory analyze what leaders *do* or are perceived by their employees. That is, they follow a behavioral approach to leadership (Antonakis and Day, 2017). The behavioral approach stands in contrast to the trait approach, which studies *traits* of leaders, such as their intelligence and personality (Judge et al., 2009). The behavioral approach to leaders analyses questions like: Do leaders provide a compelling vision (linked to transformational leadership)? Does giving clear directions improve performance (task-oriented leadership)?

The behavioral approach to leadership started with the Ohio State and the University of Michigan studies identifying two influential leadership behaviors (Antonakis and Day, 2017). The first important leadership behavior is oriented toward a person and is known as *consideration*. Considerate leaders support employees by asking them how they are, responding to them when they ask for help, and complimenting them when they do a good job. The second important leadership behavior is *initiating structure*. This task-oriented behavior is about organization: leaders who score high on initiating structure set yearly and monthly goals with employees, they highlight which activities are essential and which should be ignored, provide feedback, and they highlight deadlines.

Various leadership concepts were influenced by the foundational behavioral leadership studies (Antonakis and Day, 2017). Such concepts include supportive leadership (Rafferty and Griffin, 2006), transformational and transactional leadership (Bass and Riggio, 2006), and abusive supervision (Tepper, 2000). As we will see below, many scholars who study leadership and JD-R theory use such leadership concepts.

## Method

We carried out an electronic search using PubMed, PsychInfo, Web of Science, and Scopus to find eligible articles on the relationship between leadership and JD-R theory. We used two dimensions in our search. The first dimension concerned leadership and holds the term [leader^*^], ^*^ meaning all suffixes of the word, such as leaders or leadership. The second dimension includes JD-R theory and includes the terms [job demand^*^], [job resource^*^], [personal resource^*^], [JD-R], [job craft^*^] and [self-under^*^]. Within each dimension, we connected terms via [OR]. Between dimensions, we used [AND]. For instance, the search line for PubMed was:

(leader^*^[Title/Abstract]) AND (job demand^*^[Title/Abstract] OR job resource^*^[Title/Abstract] OR personal resource^*^[Title/Abstract] OR job craft^*^[Title/Abstract] OR self-underm^*^[Title/Abstract] OR JD-R^*^[Title/Abstract] “2001”[Date - Publication]: “2019/05”[Date - Publication])

Besides, we have asked six work and organizational psychology experts to check our list of eligible articles.

We must acknowledge limitations. We could have missed studies dedicated to leadership and JD-R theory because scholars used different terminology, such as management instead of leadership, or mentioned a job resource (like autonomy) but did not link it to JD-R theory. Furthermore, we focused on the academic published literature, leading to publication bias (Van Aert et al., 2019). However, we do not focus on effect sizes or significance levels but on general methods and conceptualizations of studies connecting leadership and JD-R theory.

### Eligibility Criteria

We included studies if they met the following criteria:

- *Type of study and participants:* The studies should focus on connecting leadership with JD-R theory.
- *Study design:* The studies should be empirical.
- *Publication status and language:* We included studies published in English peer-reviewed articles.
- *Year of publication:* Studies reported in articles published or on advance online access from 2001 onwards as in 2001, the core article on JD-R theory (Demerouti et al., 2001) was published. We ended the search on June 1, 2019.

### Literature Search

**Figure 2:**
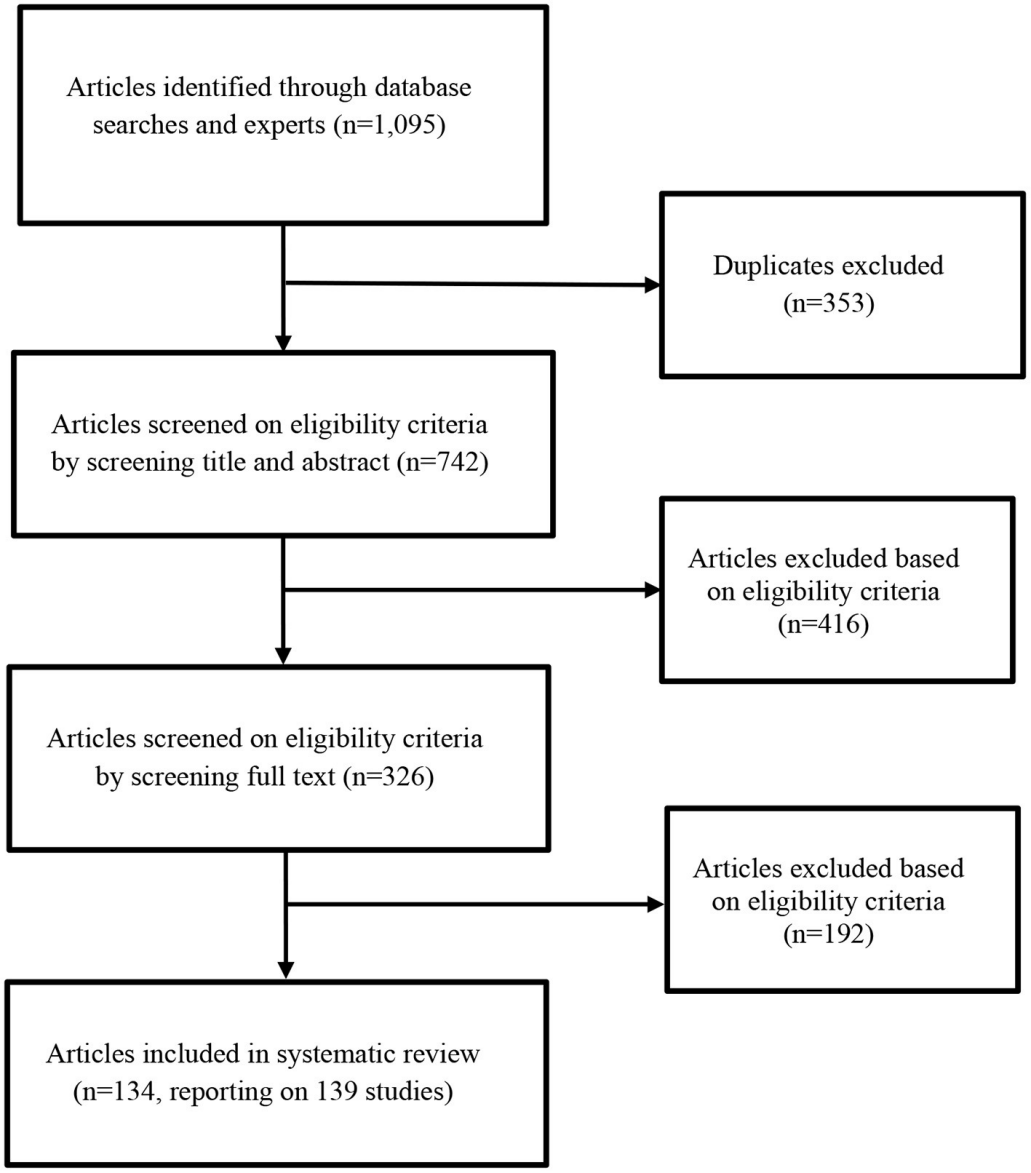
PRISMA flow chart for identifying eligible articles.

After searching, we examined the list of potentially eligible articles. Figure 2 shows the flow chart. In the end, 134 articles remained. These articles report on 139 studies, as five articles included two studies.

We noted the authors, title, journal, year, the number of studies in the article, method, design, source of data, leadership concepts, and the proposed leadership JD-R relations for each included study. Our data and codebook are available on the Open Science Framework: https://osf.io/fwgmz/.

## Results

### Method and Design

Before discussing the review's substantive results—such as the leadership concepts used and how scholars have connected leadership to JD-R theory—we look at the methods and designs (RQ1). Studying the methods and designs helps us uncover methodological strengths and weaknesses and provides directions for future studies.

Scholars predominantly used surveys (93% of all studies, see Table 1) to study the relationship between leadership and JD-R theory. For instance, Füllemann et al. (2016) studied surveys filled in by over 1,200 Swiss employees. When the employees stated that they felt that their managers supported them, they felt more engaged. A survey's benefit is evident: you can use it to collect data from many people in a structured way at low costs. However, the dominance of surveys has downsides. Surveys can suffer from social desirability bias; people fill out what they think is acceptable. For example, a person might feel that her supervisor is unsupportive but clicks “highly supportive” in the questionnaire.

**Table 1:** Methods used to study leadership and JD-R.

**Method**	**Number/percentage of studies**
**Single method**	**131 (94%)**
Survey	129 (93%)
Interviews	2 (1%)
**Mixed method**	**8 (6%)**
Survey and administrative data	6 (4%)
Survey, administrative data, and interviews	1 (1%)
Interviews and document analysis	1 (1%)
**Total**	**139 (100%)**

Two studies (1%) used interviews instead of surveys (Guglielmi et al., 2013; Muller et al., 2018). However, the interviews were just surveys in the spoken form: the authors asked people in an interview to answer from a survey-type questionnaire. Eight studies (6%) combined methods. Such mixed methods designs are beneficial as methods can complement each other (Johnson and Onwuegbuzie, 2004). For instance, Corin and Björk (2016) used interviews and documents to analyze school managers' job demands and resources. They showed that managers often lacked support from their supervisors, which resulted in increased job demands, such as huge workloads that made the managers work overtime. The scholars used illustrating quotes to substantiate the results from their document analysis.

In addition to analyzing the methods, we examined the designs. We focus on whether the designs were cross-sectional and used multiple sources, as shown in Tables 2, 3. Both aspects are critical in work and organizational psychology (Podsakoff et al., 2003; Taris and Kompier, 2003).

**Table 2:** Designs and sources used to study leadership and JD-R.

**Design**	**Number/percentage of studies**
**Cross-sectional**	**103 (74%)**
Single source	81 (58%)
Multiple sources	22 (16%)
**Longitudinal**	**36 (26%)**
Single source	28 (20%)
Multiple sources	8 (6%)
**Total**	**139 (100%)**

**Table 3:** Type of sources used in designs.

**Design**	**Number/percentage of studies**
**Single source**	**109 (78%)**
Employees	109 (78%)
**Multiple sources**	**30 (22%)**
Employees and supervisors	21 (15%)
Employees and administrative data	7 (5%)
Employees and colleagues	2 (1%)
**Total**	**139 (100%)**

The majority of studies (74%) used a cross-sectional design. Such designs are ill-suited for causal inference. Longitudinal designs (26%) can better demonstrate causality and study long-term consequences of work stressors, although they can still suffer from endogeneity (Taris and Kompier, 2003).

Of those who did use a longitudinal design, there was a wide variety. Some—such as Chen et al. (2018)—collected their data months apart. Such a design helps to uncover the long-term effects of leadership strategies. Others used short time frames. For example, Breevaart et al. (2014) used a sample of naval cadets who filled out a diary questionnaire for 34 days. Diary studies are helpful as a leader can be inspiring in general but uninspiring on a particular day. Scholars can study how such a bad day affects how followers feel and behave. For example, Breevaart et al. concluded that cadets were more engaged on days that their leader showed transformational leadership and provided contingent rewards, an aspect of transactional leadership.

Other scholars used before and after studies to test whether training employees benefits employees or the organization (for instance Van Den Heuvel et al., 2015). Such studies are valuable as they can test whether interventions to boost resources, reduce demands, or increase job crafting are related to positive or negative outcomes. However, as the studies are not randomized controlled trials. We do not know whether other factors impact the results. In other words, also these studies suffer from endogeneity (Antonakis et al., 2010).

Looking at common-source bias next, Podsakoff et al. (2003) noted that common-source bias occurs as people try to be consistent in their answers. For example, people responding to surveys might search for similarities in the questions, thereby producing relationships that do not exist at the same level in real-life settings. Using multiple sources solves this problem. Of the 139 studies included, 109 used one source (78%). Of the single source studies, all used employees as their source. Multiple source studies used employees in combination with supervisor surveys (15%), administrative data (5%), or surveys from colleagues (1%).

Concluding, most studies are survey-based, cross-sectional, single-source studies. We will show future research suggestions related to the challenges accompanying this research method and design in the discussion.

### Leadership Concepts

We will now move to the substantive topics: leadership and JD-R theory. First, we will analyze which leadership concepts scholars have used when studying leadership and JD-R theory (RQ2). In Table 4, we provide an overview of the leadership concepts that scholars used.

**Table 4:** Types of leadership concepts used.

**Type of leadership**	**Number/percentage of studies**
Transformational leadership	38 (21%)
LMX	31 (17%)
Supervisor support	21 (12%)
Servant leadership	10 (6%)
Empowering leadership	8 (4%)
Authentic leadership	8 (4%)
Quality of leadership	7 (4%)
Transactional leadership	5 (3%)
Supervisory coaching	4 (2%)
Health-promoting leadership	4 (2%)
Abusive supervision	4 (2%)
Laissez-faire leadership	3 (2%)
Fair leadership	3 (2%)
Other, <3 mentions	33 (18%)
**Total**	**179 (100%)**

*Total is more than the number of studies as studies contain more leadership concepts*.

Table 4 shows that transformational leadership is most often studied. We could have expected this, as transformational leadership is one of the most popular leadership concepts (Bass and Riggio, 2006). Transformational leaders aim to motivate employees to transcend their self-interest for the sake of the organization. They change people by developing an inspiring vision, sharing this vision, and sustaining it. A prime example of delivering an inspiring vision is the “I have a dream” speech by Martin Luther King.

Transformational leadership is intuitively appealing and inspiring visions genuinely move people (Dvir et al., 2002). However, scholars (for instance Van Knippenberg and Sitkin, 2013) have criticized the measurement of transformational leadership via the Multifactor Leadership Questionnaire. Van Knippenberg and Sitkin argue that the four transformational leadership dimensions, as measured with the Multifactor Leadership Questionnaire, are hard to distinguish from related leadership concepts such as transactional leadership, especially the contingent reward dimension of transactional leadership. The Multifactor Leadership Questionnaire critique is essential, as various studies in our review use this popular measurement tool (for instance Yizhong et al., 2019). Fortunately, scholars have developed improved measurement instruments that stay closer to the core of transformational leadership (for instance Jensen et al., 2019).

LMX is the second most often studied leadership concept. LMX describes the relationship quality between a leader and a team member (Graen and Uhl-Bien, 1995). In a situation of low Leader-Member Exchange, the leader and the member have an economic relationship. The leader pays the member, and the member works for this payment—quid pro quo. High-quality LMX relationships are different: leaders and employees trust each other and are willing to go the extra mile. However, like transformational leadership, the LMX concept is problematic. In a recent review, Gottfredson et al. (2020) provide an overview of these problems, including measurement issues, endogeneity threats, and conceptual overlaps. We advise scholars interested in studying leadership-follower relationships—which are fundamentally important—to take stock of this critique.

In addition to showing the prevalence of criticized concepts like transformational leadership and LMX, a second point that stands out is that most leadership concepts are positive. Eighty-nine percentage of the studies used positive leadership concepts. Examples are LMX, servant leadership, and ethical leadership. Only 6% was negative, for instance, studies on abusive supervision, passive leadership, and destructive leadership. We classified 4% as mixed, such as directive and paternalistic leadership. Focusing only on the positive aspects of leadership risks ignoring how leadership can be detrimental for employees. There are various ways in which leadership can negatively affect employees (Krasikova et al., 2013). For instance, one of the studies in the review (Aasland et al., 2009) shows that destructive leadership is quite widespread, as 84% percent of the surveyed employees in their sample reported exposure to destructive leadership, although much less (34%) reported exposure to at least one destructive leadership behavior often or nearly always in the last 6 months. In the discussion, we will discuss future research suggestions related to the leadership concepts used.

### Connecting Leadership and Job-Demands Resources Theory

The final research question focuses on how scholars have connected leadership and the JD-R theory. Table 5 provides an overview.

**Table 5:** Overview of connections between leadership and job demands, resources, and job crafting of employees.

**Connection**	**Number/percentage of studies**
**Leadership is resource or demand**	
Leadership = job resource	82 (41%)
Leadership = job demand	3 (3%)
**Leadership influences resources or demands**	
Leadership→ job resources	35 (17%)
Leadership→ job demands	18 (9%)
Leadership→ personal resources	12 (6%)
**Leadership influences job crafting or self-undermining**	
Leadership→ job crafting	16 (8%)
**Leadership moderates resources or demands**	
Leadership moderates job demands	19 (9%)
Leadership moderates job resources	3 (1%)
Leadership moderates personal resources	2 (1%)
**Other**	
Job demands moderate leadership	3 (1%)
Job resources→ leadership	2 (1%)
Job crafting→ Leadership	1 (<1%)
Personal resources→ leadership	1 (<1%)
Job resources moderate leadership	1 (<1%)
**Total**	**202 (100%)**

*Total is more than the number of studies as various studies contain more than one link between leadership and job demands, resources, and job crafting of employees*.

Table 5 shows a large variety of ways in which scholars have connected leadership concepts and JD-R theory. The most often used connection is conceptualizing leadership as a job resource (41%). A telling example is by Macgregor and Cunningham (2018, p. 318), who note that “job resources serve to achieve work goals and to reduce the effects of job demands, and include job security, career opportunities, supervisor, and co-worker support.” Some see leadership as a higher-order job resource different from individual-level resources like job security or autonomy. For example, Chen et al. (2018) view leadership support climate as a unit-level resource, and Mazzetti et al. (2019) view transformational leadership as an organizational resource. A few studies conceptualize leadership as a job demand (3%). Such studies often analyze negative leadership concepts, such as abusive supervision (Perko et al., 2017) or tyrannical leadership (Nielsen et al., 2018).

However, not all studies see leadership as either a job resource or a job demand. Some argue that leadership is distinct from resources and demands. Leadership impacts job resources (17%), job demands (9%) or related JD-R concepts like personal resources (6%). For example, Fernández-Muñiz et al. (2017, p. 405) argue that job demands and resources are “conceivably conditioned by managers' decisions and policies because the managers have the power to define the demands and resources of the job.” Other studies focus on the impact of leadership on job crafting (8%). Such studies often use autonomy-supporting leadership concepts like servant leadership (Yang et al., 2017), engaging leadership (Mäkikangas et al., 2017), or empowering leadership (Thun and Bakker, 2018). These studies often conclude that leaders can help employees to craft their jobs.

In addition to directly impacting job demands, resources, and job crafting, leadership can be a moderator. It can change the impact of job demands (9% of studies), job resources (1%), or personal resources (1%). Such moderating effects are interesting to study when working conditions are hard to change. For instance, Breevaart and Bakker (2018) showed that transformational leadership moderates the effects of hindrance and challenge demands on employee work engagement. Transformational leadership boosted work engagement on days characterized by high challenge job demands and protected work engagement on days characterized by high hindrance job demands.

### Three Main Connections Between Leadership and JD-R Theory

It is clear from the preceding analysis that scholars have connected leadership and the JD-R theory in various ways. We found 14 potential connections. In addition, Table 4 shows that there is a host of leadership concepts used. Both outcomes of our review are understandable. It connects with the JD-R theory's heuristic and flexible nature (Schaufeli and Taris, 2014; Bakker and Demerouti, 2017). In JD-R theory, there are no specific concepts linked to each other, such as with the job-demand control model. Instead, the JD-R theory is flexible. In this way, the JD-R theory can be applied in many situations. However, as Bakker and Demerouti (2017, p. 278) note, this flexibility could be the Achilles' heel of the theory, reducing the specificity and the quality of its predictions. Relating this to the topic of this review, we note that leadership can be integrated into the JD-R theory, but there is no straightforward way to integrate it. There are many possibilities. This makes it hard to build upon each other's work. For cumulative knowledge to develop, we should conceptualize how leadership and JD-R theory can be connected.

We, therefore, provide a new, hopefully valuable, theoretical perspective on how leadership and JD-R theory can be connected. We see leadership as a construct located on a higher level than the dimensions of JD-R theory. Our review found various studies that use a similar conceptualization (like Bernstrom and Kjekshus, 2012; Fernet et al., 2015; Schaufeli, 2015). Organizations are composed of various levels (Kozlowski and Klein, 2000; Bakker and Demerouti, 2018). Most JD-R studies have investigated processes at the individual, employee, level. Hence, employees report their job demands, resources, engagement, and burnout (for instance Salanova et al., 2013). Employees themselves may also alter their working demands and resources via job crafting (Wrzesniewski and Dutton, 2001). Next to such individual approaches, leaders—situated at higher organizational levels—can take action. If they know which job demands and resources need attention; they can take measures so that such working conditions are improved.

**Figure 3:**
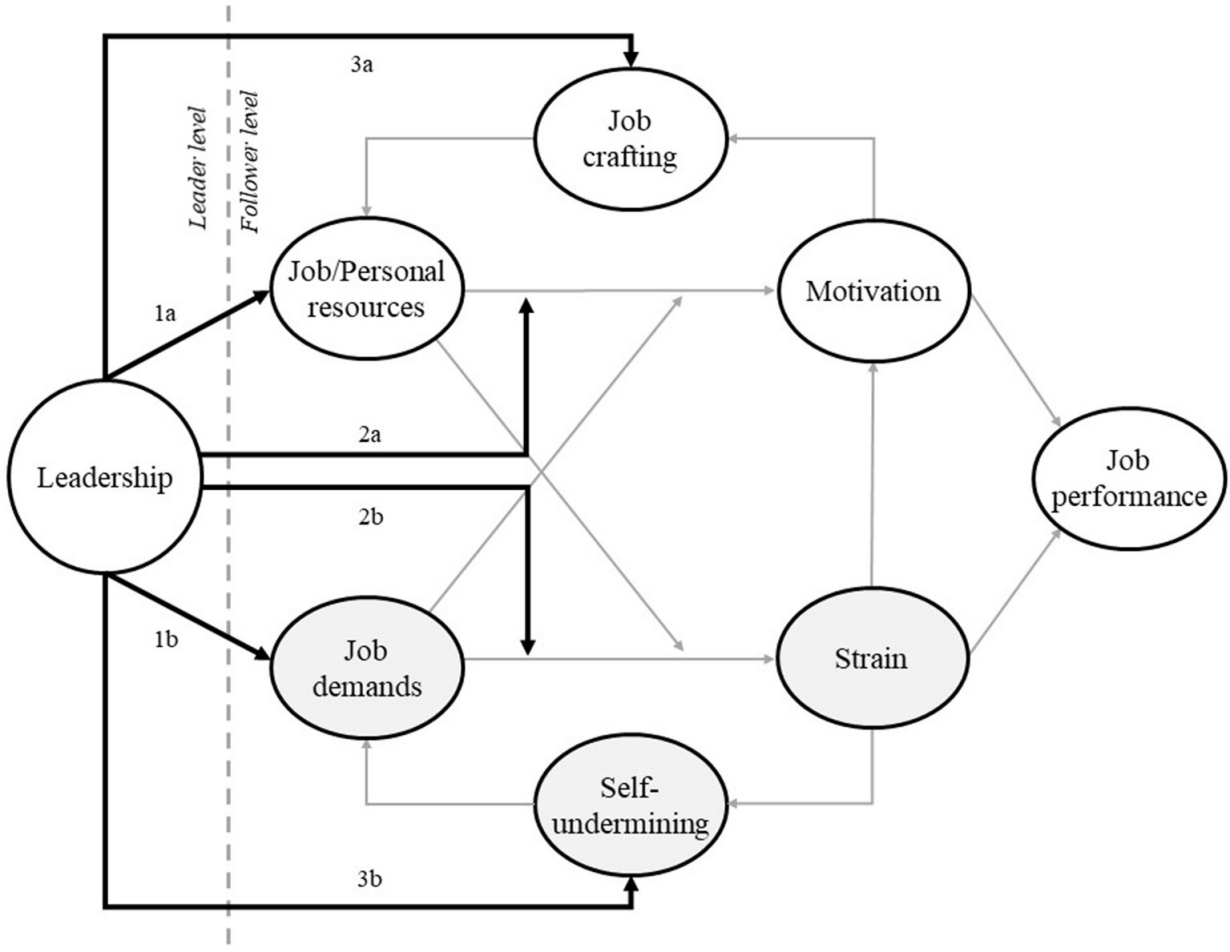
Connecting leadership and Job Demands–Resources (JD-R) theory. Leadership impacts JD-R in three distinct ways.

In Figure 3, we highlight that leadership may impact the JD-R theory in three distinct ways. There are other ways in which leadership and JD-R theory can be connected, as discussed above. However, by highlighting three ways, we develop a simple model. Goldstein and Gigerenzer (2011, p. 392) state, “the beauty of simple models is that one can easily discover their limits, that is, their boundary conditions, which in turn fosters clarity and progress.”

First, leadership can directly impact job demands, job resources, and personal resources (see Figure 3, paths 1a and 1b). A clear example is servant leadership. A servant leader can increase job resources like autonomy and social support, which leads to higher motivation and performance (Chiniara and Bentein, 2016). Furthermore, leaders can decrease job demands. Fernet et al. (2015) showed that transformational leadership reduces cognitive, emotional, and physical job demands, thereby reducing psychological strain. However, leadership may also increase job demands. Molino et al. (2019) showed that destructive leadership increases workload, which subsequently increases workaholism and exhaustion.

Second, leadership can moderate the link between job/personal resources and motivation (path 2a) and the link between job demands and strain (path 2b). Leadership can, for instance, strengthen the effects of personal resources. Leadership may help employees by stimulating them to use their resources, such as their proactive personality, which ultimately could increase engagement (cf. Caniëls et al., 2018). Furthermore, leadership can reduce the effects of high job demands. For instance, a servant leader may make it easier for employees to deal with job demands like high workload by providing employees with autonomy. Hence, maybe the leader may not be able to reduce the workload, but she can help by giving employees more autonomy to deal with this it. Furthermore, leaders can provide a compelling and meaningful vision with which employees identify. Such an inspiring vision helps employees feel that a high workload is worth it: they are motivated to work for a good cause (Grant, 2007).

Third and finally, leadership can directly influence follower job crafting (path 3a) and follower self-undermining (path 3b). Job crafting is a crucial self-reinforcing path in JD-R theory. People craft their jobs when they proactively change their job demands and resources. Wang et al. (2017) and Thun and Bakker (2018) showed that empowering leadership positively relates to follower job crafting. When leaders empower their employees, these employees are more inclined to optimize their work environment. By stimulating job crafting, leaders could help followers to stay engaged at work. However, leaders can also increase self-undermining. For instance, when abusive supervision increases employees' mistakes, the employee needs to correct these mistakes, adding to the already high job demands (Bakker and Costa, 2014).

## Discussion

This article set out to perform a systematic literature review to answer questions about leadership and JD-R theory. We analyzed the methods and designs (RQ1), the leadership concepts (RQ2), and the ways scholars have connected leadership and JD-R theory (RQ3). We identified 139 studies that investigated leadership and JD-R theory. The findings suggest that leaders may influence employees in various ways. This multitude of potential connections shows the heuristic nature of the JD-R theory. However, it also makes it hard to develop cumulative knowledge. We have therefore structured this in three primary connections: (1) leadership can directly impact job demands, job resources, and personal resources, (2) leadership can moderate the link between job/personal resources and motivation as well as the link between job demands and strain, and (3) leadership can directly impact follower job crafting and self-undermining.

### Future Research Directions

Our review also identified gaps in the literature. We will discuss these gaps below and offer an agenda for future research on leadership and JD-R theory. To start, most scholars use cross-sectional surveys to study leadership and JD-R theory. Cross-sectional surveys help when we want to measure the prevalence of leadership behavior (for instance Aasland et al., 2009). However, we found that scholars also used cross-sectional survey designs to establish cause-and-effect relationships. Cross-sectional designs are ill-suited for this (Antonakis et al., 2010). A suggestion for future research is to take causal inference seriously. Various approaches can be used, including field experiments, regression discontinuity designs, and natural experiments. Such econometric designs are new for many work and organizational psychologists, but accessible overviews are available (Antonakis et al., 2010; Sieweke and Santoni, 2020). We urge scholars to check these out.

A classic example of a study that takes causal inference seriously is by Dvir et al. (2002). Dvir et al. randomly assigned military leaders to either a transformational leadership training or an eclectic leadership training. The results showed that the leaders in the transformational group had a more positive impact on the development of their direct followers and on the performance of their indirect followers than leaders in the eclectic leadership group. Such leadership experiments conducted in the field can be beneficial to test which leadership training is beneficial to improve the job design of employees. For instance, scholars could investigate which leadership approach is most beneficial to increase job crafting. They could pit servant leadership against transformational leadership and assign leaders randomly to trainings were these leadership concept are taught. A few months later, they could measure the performance and well-being of the employees these leaders supervise. Which training would be most beneficial? Future research without endogeneity problems is paramount to develop rigorous and relevant research on leadership and JD-R theory.

We will now analyze future research suggestions regarding theory development. First, regarding leadership concepts, we found that scholars use leadership concepts like transformational leadership and LMX. As noted, scholars are critical of these and similar concepts, especially how they are currently measured (Yukl, 1999; Van Knippenberg and Sitkin, 2013; Gottfredson et al., 2020). Scholars aiming to use these concepts should analyze these critiques in detail. Sometimes these concepts can be helpful, but proper measurements should be used. For instance, high-quality measurement instruments of transformational leadership have been developed (Jensen et al., 2019). However, sometimes scholars should search for other concepts, as argued regarding LMX (Gottfredson et al., 2020).

Furthermore, it is essential to theorize the mechanisms underlying the relationships between leadership and JD-R theory (Schaufeli and Taris, 2014; Bakker and Demerouti, 2017). For instance, scholars can use self-determination theory (Ryan and Deci, 2000) or the conservation of resources theory (Hobfoll, 1989) to explain *why* certain leadership behaviors are beneficial. Furthermore, the self-expansion model can be used to analyze how leadership affects employees. Simply put, the model posits that people aim to expand their potential efficacy and that a way they do this is via close relationships (Aron et al., 2004). Dansereau et al. (2013) connect the self-expansion model with leadership, showing that self-expansion can happen when leaders and employees form close relationships. As self-expansion is about increases resources via someone else—in this case, the leader—the model seems valuable for studying how leadership affects the job resources of employees.

Especially longitudinal studies can help study the mechanisms involved. Various have already been conducted, with time lags spanning from days (Tims et al., 2011), weeks (Bennett et al., 2016), months (Bernstrom and Kjekshus, 2012), to even years (Nielsen et al., 2019). Future studies can take stock of valuable overviews regarding process and time in leadership (Fischer et al., 2017; Mcclean et al., 2019) and the JD-R literature (Lesener et al., 2019). For instance, Mcclean et al. move beyond a discussion on time lags. Instead, they provide a nuanced conceptualization of time. They distinguish between sudden shifts (such as the death of leaders), gradual growth and decay (like the development of social support received from supervisors over years in your job), and ebb and flow (for instance a leader who is strict on Mondays but relaxed on Fridays).

A final suggestion focuses on the negative aspects of leadership. Positive leadership concepts such as transformational and supervisory support are dominant. Future studies could look at the negative aspects of leadership, such as analyzing how leaders reduce job resources or studying when certain leadership behaviors may be “too much of a good thing” (Pierce and Aguinis, 2013). There is extensive literature on negative leadership concepts that scholars interested in leadership and JD-R theory can draw upon (for instance Einarsen et al., 2007; Fischer et al., 2021).

### Implications for Practitioners

By highlighting three ways in which leadership can impact employees' job characteristics, we hope to broaden the perspective of leaders on how to improve the work lives of employees. First, leaders can aim to influence job demands and resources directly. For instance, when employees experience a high workload, leaders can help reduce this job demand by deciding what the priorities are for the organization and which work aspects can safely be ignored (Hesselgreaves and Scholarios, 2014). Leaders can also increase job resources. For instance, leaders can increase job autonomy by letting employees decide when and where to work (Gajendran and Harrison, 2007).

However, sometimes leaders cannot change job demands and resources directly. Such situations often occur for mid-level leaders. However, even in such cases, leaders can be helpful. Hence, the second way in which leaders can be influential is by moderating job demands and resources. For instance, Syrek et al. (2013) showed that transformational leadership lowered the impact of time pressure on work-life balance and exhaustion. They conclude that transformational leadership is important for employee work–life balance and exhaustion when time pressure is high. Hence, even when leaders cannot influence a job characteristic—in this case, time pressure—they can buffer the effects of such a job characteristic.

The third way in which leadership can affect job characteristics of employees is by influencing job crafting and self-undermining. For instance, leaders can aim to make it easier for people to craft their jobs. When leaders give autonomy to their employees and are open about their own weaknesses, employees can feel the freedom to seek new challenging projects, learn new skills, and ask for feedback (Harju et al., 2018). However, leaders should be aware that the ways employees crafts their jobs are in line with organizational requirements and whether colleagues see the job crafting as legitimate (see also Hornung et al., 2010). Job crafting has its boundaries.

## Conclusion

There is an abundance of studies on the connections between leadership and JD-R theory. We hope to contribute to the literature by presenting a systematic review of these studies and developing ways future research suggestions to improve the field. We identified three ways in which leadership can influence the elements of JD-R theory. Leadership can (1) directly impact job demands, job resources, and personal resources, (2) moderate the link between job/personal resources and motivation as well as the link between job demands and strain, and (3) directly impact follower job crafting and self-undermining. We also indicated methodological and theoretical research gaps that scholars can address to take the field forward. These include strengthening research designs, using proper measurements of leadership concepts, clarifying the mechanisms underlying the connections between leadership and JD-R, and researching negative leadership concepts. Studying leadership and JD-R theory should prove to be a timely and productive endeavor for researchers and practitioners alike.

## Data Availability Statement

Our data and codebook are available on the Open Science Framework: https://osf.io/fwgmz/.

## Author Contributions

LT conducted the data analysis. LT and AB wrote the manuscript. All authors contributed to the article and approved the submitted version.

## Conflict of Interest

The authors declare that the research was conducted in the absence of any commercial or financial relationships that could be construed as a potential conflict of interest.

## Publisher's Note

All claims expressed in this article are solely those of the authors and do not necessarily represent those of their affiliated organizations, or those of the publisher, the editors and the reviewers. Any product that may be evaluated in this article, or claim that may be made by its manufacturer, is not guaranteed or endorsed by the publisher.
